# Multimodal hypoxia imaging and intensity modulated radiation therapy for unresectable non-small-cell lung cancer: the HIL trial

**DOI:** 10.1186/1748-717X-7-157

**Published:** 2012-09-14

**Authors:** Vasileios Askoxylakis, Julien Dinkel, Monika Eichinger, Bram Stieltjes, Gregor Sommer, Ludwig G Strauss, Antonia Dimitrakopoulou-Strauss, Annette Kopp-Schneider, Uwe Haberkorn, Peter E Huber, Marc Bischof, Jürgen Debus, Christian Thieke

**Affiliations:** 1 Department of Radiation Oncology, University of Heidelberg, INF 400, 69120, Heidelberg, Germany; 2 Clinical Cooperation Unit Radiation Oncology, German Cancer Research Center (DKFZ), INF 280, 69120, Heidelberg, Germany; 3 Department of Radiology, German Cancer Research Center (DKFZ), INF 280, 69120, Heidelberg, Germany; 4 Quantitative Imaging-based Disease Characterization, German Cancer Research Center (DKFZ), INF 280, 69120, Heidelberg, Germany; 5 Clinical Cooperation Unit Nuclear Medicine, German Cancer Research Center (DKFZ), INF 280, 69120, Heidelberg, Germany; 6 Department for Biostatistics, German Cancer Research Center (DKFZ), INF 280, 69120, Heidelberg, Germany; 7 Department of Nuclear Medicine, University of Heidelberg, INF 400, 69120, Heidelberg, Germany

**Keywords:** Hypoxia, Imaging, Radiotherapy, Non-small-cell lung cancer

## Abstract

**Background:**

Radiotherapy, preferably combined with chemotherapy, is the treatment standard for locally advanced, unresectable non-small cell lung cancer (NSCLC). The tumor response to different therapy protocols is variable, with hypoxia known to be a major factor that negatively influences treatment effectiveness. Visualisation of tumor hypoxia prior to the use of modern radiation therapy strategies, such as intensity modulated radiation therapy (IMRT), might allow optimized dose applications to the target volume, leading to improvement of therapy outcome. ^18^ F-fluoromisonidazole dynamic positron emission tomography and computed tomography (^18^ F-FMISO dPET-CT) and functional magnetic resonance imaging (functional MRI) are attractive options for imaging tumor hypoxia.

**Methods/design:**

The HIL trial is a single centre study combining multimodal hypoxia imaging with ^18^ F-FMISO dPET-CT and functional MRI, with intensity modulated radiation therapy (IMRT) in patients with inoperable stage III NSCLC. 15 patients will be recruited in the study. All patients undergo initial FDG PET-CT and serial ^18^ F-FMISO dPET-CT and functional MRI before treatment, at week 5 of radiotherapy and 6 weeks post treatment. Radiation therapy is performed as inversely planned IMRT based on 4D-CT.

**Discussion:**

Primary objectives of the trial are to characterize the correlation of ^18^ F-FMISO dPET-CT and functional MRI for tumor hypoxia imaging in NSCLC and evaluate possible effects of radiation therapy on tumor re-oxygenation. Further objectives include the generation of data regarding the prognostic value of ^18^ F-FMISO dPET-CT and functional MRI for locoregional control, progression free survival and overall survival of NSCLC treated with IMRT, which will form the basis for larger clinical trials focusing on possible interactions between tumor oxygenation and radiotherapy outcome.

**Trial registration:**

The ClinicalTrials.gov protocol ID is NCT01617980

## Background

Lung cancer is the leading cause of cancer mortality. The disease is worldwide diagnosed in about 1.35 million patients yearly and is responsible for about 1.18 million deaths yearly [1]. Non-small-cell lung cancer (NSCLC) accounts for 75% of all cases. The treatment of choice is surgery however a radical resection is possible in only 20% of all cases. Radiation therapy alone or combined with chemotherapy are therapeutic alternatives for tumors that are surgically not resectable. Application of 60-66 Gy by external beam radiotherapy results in a mean local tumor control of about 12 months [2], whereas combined radio-chemotherapy, preferably platinum-based, leads to a significant survival improvement compared to irradiation alone [3].

Although there is evidence that dose escalation is related with increased local control and improved overall survival, a higher radiation dose is also related with increased lung toxicity and severe side effects, such as radiation induced pneumonitis and lung fibrosis [4]. To reduce side effects, advanced technologies that allow a more accurate dose delivery to the target volume minimizing healthy tissue toxicity have been developed. A promising technology in this respect is the intensity modulated radiation therapy (IMRT) [5].

Furthermore, use of fluorine-18 deoxyglucose dynamic positron emission tomography and computed tomography (FDG dPET-CT) improved disease staging, whereas the development of four-dimensional computed tomography (4D-CT) enabled a more precise target volume definition [6,7]. 4D-CT can improve radiotherapy targeting, since it provides information about the motion of the target but also about changes of the pulmonary parenchyma as a result of breathing-associated changes in air content [8,9]. In addition, advanced dynamic contrast enhanced magnetic resonance imaging (DCE-MRI) techniques allow a better visualization of tumor heterogeneity, as well as an improved assessment of tumor vascularization and more accurate differentiation between benign processes such as inflammation or atelectasis and malignant tumor lesions [10,11].

However, despite the development of diagnostic and therapeutic modalities, lung tumors still show a variable resistance to available regimens of radio-chemotherapy. One of the factors that increase therapy resistance is hypoxia. Tumor hypoxia is associated with a malignant phenotype, characterized by high invasiveness, increased potential for progression and metastasis and poor prognosis [12,13]. Subphysiologic levels of oxygen are present in the majority of human tumors and lead to an up to 3-fold increase of resistance against antineoplastic strategies [14].

The leading role of hypoxia in radiation resistance reveals the necessity for the development and evaluation of hypoxia imaging assays. Such assays would allow a better characterization of tumor heterogeneity and facilitate the improvement of targeted therapies, as well as the development of novel strategies for prediction of treatment outcome. An extensively investigated tracer for visualization of tumor hypoxia by dynamic positron emission tomography and computed tomography (dPET-CT) is fluorine-18-labeled fluoromisonidazole (^18^ F-FMISO; half time 110 min). Various preclinical and clinical studies have revealed a correlation between oxygen measurements and ^18^ F-FMISO uptake [15]. In metastatic head and neck cancer, the retention of ^18^ F-FMISO was found to be significantly greater in hypoxic tumors, especially at pO_2_ values <5 mmHg [16]. A study of 12 patients with head and neck carcinoma, who received FMISO-PET scans before radiotherapy, revealed that the tracer uptake was a prognostic indicator of treatment response [17]. In regard to NSCLC, a study performed in 14 patients demonstrated an association between high tumor-to-muscle FMISO uptake ratios and risk of relapse [18]. Furthermore, a trial of 8 patients with NSCLC who were treated by combined radio-chemotherapy and received serial ^18^ F-FMISO-PET scans showed an association between FMISO uptake decrease post treatment and favourable therapy outcome [19].

Further non-invasive methods for *in vivo* oxygenation monitoring include modern magnetic resonance imaging (MRI) and magnetic resonance spectroscopy (MRS) applications. MRI approaches comprise perfusion functional MRI for evaluation of tissue hemodynamics through characterization of blood flow patterns, as well as approaches based on the effects of local oxygen tension on the magnetic susceptibility effects of oxy- and deoxyhemoglobin and on the effects of paramagnetic oxygen on the relaxation times of tissue water [20].

Aim of the present trial is to investigate the correlation of ^18^ F-FMISO dPET-CT and functional MRI for tumor hypoxia imaging in patients with inoperable stage III NSCLC, treated with 4D-CT based IMRT. Furthermore, through serial ^18^ F-FMISO dPET-CT and functional MRI investigations prior, during and post treatment, possible effects of radiation therapy on tumor re-oxygenation and their influence on treatment response will be evaluated.

## Methods/design

### Trial organisation

The HIL-trial has been designed by the study initiators at the Clinical Cooperation Unit Radiation Oncology at the German Cancer Research Center (DKFZ), the Clinical Cooperation Unit Nuclear Medicine at DKFZ, the Department of Radiology at DKFZ and the Department of Radiation Oncology at the University of Heidelberg. The trial is carried out at DKFZ in co-operation with the Department of Radiation Oncology at the University of Heidelberg.

### Coordination

The trial is coordinated by the Clinical Cooperation Unit Radiation Oncology at DKFZ and the Department of Radiation Oncology at the University of Heidelberg. DKFZ is responsible for trial management and quality assurance including reporting and database management.

### Study design

**Figure 1:**
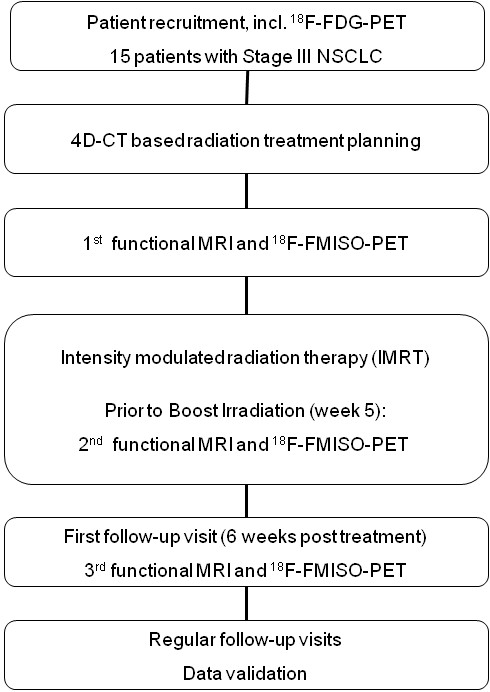
Trial workflow.

The study is designed as a single centre trial with an accrual of 15 patients with inoperable stage III NSCLC. Patients fulfilling the inclusion criteria are treated with intensity modulated radiation therapy (IMRT). All patients undergo serial ^18^ F-FMISO dPET-CT and functional MRI before treatment, at week 5 of radiotherapy and 6 weeks post treatment. The trial workflow is depicted in Figure 1.

### Study objectives

Primary aim of the study is to characterize the correlation of ^18^ F-FMISO dPET-CT and functional MRI for tumor hypoxia imaging in patients with stage III NSCLC treated with intensity modulated radiation therapy (IMRT). This will be achieved through correlation between functional MRI parameters, such as diffusion coefficients and ^18^ F-FMISO dPET-CT parameters, such as standard uptake value (SUV) in matched regions of interest. Further objectives are to evaluate changes in ^18^ F-FMISO dPET-CT and functional MRI parameters during radiation treatment and characterize their prognostic value for locoregional control, progression free survival and overall survival.

### Investigators

Patient treatment is performed by radiation oncologists at the Clinical Cooperation Unit Radiation Oncology at DKFZ. 4D-CT and functional MRI scans are performed at the Department of Radiology at DKFZ. dPET-CT studies are carried out at the Clinical Cooperation Unit Nuclear Medicine at DKFZ.

### Data handling, storage and archiving

All findings, including clinical and laboratory data, are documented by the investigator or an authorized member of the study team in the subject’s medical record. The investigator is responsible for ensuring that all sections are completed correctly and that entries can be verified against source data. All missing data or inconsistencies are reported back to the investigators and clarified by the responsible investigator. If no further corrections are to be made in the database it will be declared closed and used for analysis. The data will be stored and archived according to §13 of the German GCP-Regulation and §28c of the German X-Ray Regulation (RöV) and §87 of the German Radiation Protection Regulation (StrlSchV) for at least 30 years after the initial termination.

### Ethics, informed consent and safety

The final protocol was approved by the ethics committee of the University of Heidelberg, Heidelberg, Germany (Nr: S-249/2009). The trial is sponsored by the Federal Ministry of Education and Research (BMBF) of Germany as part of the research fund “DOT-MOBI” (Nr.: 01IB08002B). This study complies with the Helsinki Declaration and its recent German version, the Medical Association code of conduct, the principles of Good Clinical Practice (GCP) and the Federal Data Protection Act. The trial is carried out in keeping with local legal and regulatory requirements. The medical secrecy and the Federal Data Protection Act are followed. The ClinicalTrials.gov protocol ID is NCT01617980.

### Patient selection

Inclusion criteria for the trial are:

Documented inoperable, histologically confirmed NSCLC stage III.

Sufficient remaining lung function (FeV_1_ > 1.5 l/s or at least 50% of the respective individual norm value).

Karnofsky Performance Score of 70% or higher.

Patients > 18 years of age.

Adequate haematological function (wbc > 3000 × 10^3^ /ml, thc >100 × 10^6^ /ml, Hb > 10 g/dl)

Adequate hepatic and renal function

Written informed consent

Exclusion criteria for the trial are:

Patient refusal

Severe concurrent systemic disease

Other malignancies

Hypersensitivity to x-ray contrast medium or ^18^ F-FMISO

Claustrophobia

Cardiac pacemaker

Severe renal or hepatic insufficiency

Pregnancy or lactation

### Study plan

15 patients are included in the study according to the criteria above. Eligible patients are informed about participation in the trial with possible benefits and risks, and written informed consent is obtained. Staging is completed through performance of thoracic CT scan, abdominal ultrasound and ^18^ F-FDG dPET-CT scan.

### IMRT treatment planning

After immobilization in a vacuum mattress a contrast-enhanced thoracic CT scan including 4D respiratory triggering is carried out. CT data are synchronized with the recorded respiratory signal and 4D reconstructions are performed to evaluate the motion of the thoracic organs and the tumor during the breathing cycle. Based on the 4D-CT data set, radiation treatment planning is performed as inverse planning. ^18^ F-FMISO dPET-CT and functional MRI data are not included in target volume definition or dose prescription.

### Radiation therapy

Radiation therapy is performed as intensity modulated radiation therapy (IMRT). A dose of 50-54 Gy is applied to the primary tumor and mediastinal lymph nodes in daily fractions of 5 × 2 Gy. Subsequently, the primary tumor and involved lymph nodes are boosted to a total dose of 60-72 Gy in daily fractions of 5 × 2 Gy. Tolerance doses of thoracic organs at risk are not exceeded.

Treatment is carried out on an out-patient basis unless the condition of the patient requires hospital administration.

### ^18^ F-FMISO positron emission tomography

^18^ F-FMISO is provided by Iason (Graz, Austria). ^18^ F-FMISO dPET-CT investigations are performed prior to radiotherapy, at week 5 of radiation therapy and at 6 weeks post treatment. dPET-CT examinations are performed after the i.v. injection of ^18^ F-FMISO using an Biograph mCT S128 (Siemens Medical Solutions Co., Erlangen, Germany). The dynamic studies are acquired with a 28-frame protocol for one hour. Quantification is performed following the iterative reconstruction of the dPET-CT data using a dedicated software package. Generally, volumes-of-interest (VOIs) are placed over the tumor and reference regions, followed by a compartment and non-compartment analysis. A two-tissue compartment model is the model of choice and five parameters are obtained. The quantification includes the calculation of the fractional blood volume, also named as vessel density (VD), the parameters k1 and k2, which reflect the influx and efflux of FMISO into and out of the cells, and k3 and k4, which are related to the trapping and re-oxygenation of FMISO. For the input function the mean value of the VOI data obtained from a large arterial vessel like the descending aorta is used. Besides the VOI based analysis, parametric images are calculated to assess dedicated parameters of the FMISO kinetics.

Furthermore, a non-compartment model based on the fractal dimension is used. The fractal dimension (FD) is a parameter for the heterogeneity and is calculated for the time activity data of each individual VOI. The values of the fractal dimension vary from 0 to 2 showing the deterministic or chaotic distribution of the tracer activity. We use a subdivision of 7 × 7 and a maximal SUV of 20 for the calculation of FD. Details of the quantification of the dPET data have been published elsewhere [21].

### Functional MRI

Functional MRI investigations are performed prior to radiation therapy, at week 5 of radiation therapy and at 6 weeks post treatment. All examinations are performed using a clinical 1.5-T MRI scanner (Magnetom Avanto, Siemens Medical Solutions, Erlangen, Germany).

The standard protocol comprises a coronal and a transversal breath-hold TrueFISP, T2w single-shot half-Fourier TSE (HASTE) and T1w 3D-GRE (VIBE) sequence. Afterwards, a navigator triggered transversal T2w-FatSat sequence (T2-FS BLADE) is carried out.

Diffusion weighted imaging (DWI) is performed using an axial single shot echoplanar (EPI) sequence with and without flow-compensation. A total of ten b-values (0, 10, 25, 50, 100, 200, 300, 400, 500 and 800 s/mm2) is acquired, enabling extraction of diffusion and perfusion parameters. DWI parameters are evaluated based on the Intravoxel Incoherent motion (IVIM) model [22], yielding the parameters perfusion fraction f and diffusion constant D, using in house developed open-source software MITK Diffusion, Version 2011 (downloadable at http://www.mitk.org). The parameter estimation is based on the assumption that the diffusion measurement is influenced by mainly two effects, a perfusion related effect introduced by the molecules moving in the capillary network (pseudodiffusion coefficient, D*) and extravascular effects of passive diffusion (D). Since a simultaneous nonlinear fit for all parameters D, D*, and the weighting coefficient f can be instable, measurement at b-values greater than 200 s/mm^2^ are used in a first step to estimate f and D as described [23]. D* is then calculated in a second step by using exhaustive search.

Lung cancer perfusion is assessed using a spoiled 3D gradient echo sequence after bolus injection of 0.07 mmol/kg body weight of Gd-DTPA. Ten acquisitions in one expiratory breath hold (10 × 2.25 s = 22 s) are followed by 50 navigator-triggered acquisitions under free breathing (total TA 4½ min). After a co-registration of the 3D data sets, a ROI-based visualization of the signal-time curves is performed as described [24].

Furthermore, a time-resolved echoshared gradient echo sequence (TWIST) is performed for assessment of three-dimensional tumor motion (240 × 0.5 s = TA 2 min). A dynamic 2D-TrueFISP sequence acquired in coronal orientation crossing the centre of the tumor provides additional information about lung and tumor motion during the breathing cycle.

Contrast-enhanced sequences breath hold 3D-GRE sequence (VIBE) completes this protocol. The in-room time for the complete protocol is approximately 30 min.

### Clinical follow up

The first follow-up is planned 6 weeks post treatment and includes study-related ^18^ F-FMISO dPET-CT and functional MRI examinations. Further regular follow-up visits are scheduled every 3 months for the first 2 years, every 6 months for the following 3 years and thereafter yearly. Individual trial participation is completed three years after patient enrolment or death of the patient.

The therapeutic efficacy will be evaluated through thoracic CT-scan at follow up visits.

### Evaluation of local response

Local response evaluation is performed according to the RECIST Criteria (Response Evaluation Criteria in Solid Tumours) [25].

### Statistical analysis

The study is a prospective and non-randomized trial with inclusion of 15 patients. Repeated examinations with ^18^ F-FMISO dPET-CT and functional MRI lead to longitudinal data for every patient. The data consists of maps obtained from both measurement devices. Data are quantitative measurements but may be dichotomized or categorized into more than two classes. For all parameters, differences between the site of local relapse and a selected control region are derived and compared by paired tests at 5% level.

All analyses are exploratory. A sample size calculation cannot be performed because neither standard deviation of the differences has been estimated before, nor the relevant difference is known. Therefore, the study will be used to generate hypotheses for future research.

In the frame of the radiation therapy planning study, virtual radiation therapy strategies will be compared to the radiation therapy administered to the patient.

## Discussion

Radiation therapy in combination with platinum-based chemotherapy is the treatment of choice for inoperable non-small-cell lung cancer. The development of modern radiation delivery techniques, such as intensity-modulated radiotherapy (IMRT) optimized treatment planning, enabled better sparring of surrounding healthy tissues and decreased treatment-related toxicity [26,27].

Compared with other cancer entities, in case of lung cancer tumor motion during the breathing cycle and variation of the target size due to changes in air content of the pulmonary parenchyma enhance the complexity of issues involved in radiation therapy planning, impede the definition of target volume and limit IMRT. In recent years, major progress has been made, mainly through the development of four dimensional respiratory triggering CT-scan planning modalities and MRI applications that allow a spatial resolution in the assessment of tumor, healthy lung parenchyma, chest wall and diaphragm [28].

The outcome of radiation therapy is however often limited by features of the tumor microenvironment. The therapeutic effect of radiation therapy is known to be negatively influenced by low tumor oxygen levels. Therefore, better understanding of the correlation between radiation resistance enhancing parameter, such as tumor hypoxia, and outcome of radiation therapy applications is of high priority for optimization of radiation therapy strategies and improvement of treatment efficacy.

In this respect, visualization of tumor heterogeneity in regard to hemodynamics and oxygen concentration is necessary. Imaging applications for hypoxia assessment include FMISO PET-CT and functional magnetic resonance imaging. The use of FMISO in hypoxia specific PET approaches is based on its selective reduction in tumor regions with decreased oxygen concentration, and the binding of its metabolites to macromolecules [15]. However, PET imaging is limited mainly through a reduced spatial resolution. MRI provides high spatial resolution structural and functional information on tumor vasculature and perfusion, including important anatomical details. Therefore, dPET-CT based investigation of variations in FMISO uptake and analysis of functional MRI parameter can provide complementary data about tumor vascularisation, microenvironment, functional and anatomical structures.

Multimodal imaging studies in animal models were combined with tissue section analysis to relate the *in vivo* non-invasive data to the tumor microenvironment. These studies demonstrated a positive correlation between perfusion related, MRI-derived parameter with early FMISO PET intensity and a negative correlation with late FMISO slope, providing evidence for the hypothesis that tumor regions with reduced perfusion are hypoxic [29]. However, the same study also revealed issues which should be considered in multimodal approaches, such as volume averaging effects in PET as a result of its lower spatial resolution and perfusion effects of FMISO accumulation in the tumor [29]. Based on these data, a clinical trial investigated tumor perfusion using MRI and hypoxia measured by FMISO-PET in 13 patients with nodal metastases of head and neck cancer [30]. The results of this study also revealed a negative correlation between FMISO uptake and the perfusion value, supporting the hypothesis that hypoxic tumors are poorly perfused [30].

Major pathophysiological mechanisms for the onset of tumor hypoxia do not only include structural and functional abnormalities of the tumor vasculature, which can be assessed by perfusion protocols, but also adverse molecular diffusion. The diffusion geometry of tumors can be assessed and visualized by diffusion-weighted MRI (DWI). DWI has been successfully applied in various clinical trials for determination and monitoring of treatment response, indicating that lesions with locoregional recurrence during follow-up correlated with significantly lower diffusion [31].

Based on the empiricism of previous trials and on the fact that, although single methods for non-invasive hypoxia imaging are well established, still their complementary potential is not utilized, we designed the HIL trial. Aim of this trial is the evaluation of multimodal tumor hypoxia imaging using ^18^ F-FMISO positron emission tomography and functional MRI in 15 patients with inoperable stage III NSCLC, receiving 4D-planned intensity modulated radiation therapy. We chose for our trial NSCLC because of the major challenges that are associated with this tumor entity, i.e. the tumor motion due to changes in air content, which impedes the exact image co-registration that is however necessary for pixel-by-pixel comparisons. The HIL trial is an exploratory study. Data gained from this pilot investigation of simultaneous, serial, multimodal approaches on hypoxia visualization will form the basis for larger clinical studies characterizing in detail possible interactions between oxygen concentration and radiation outcome.

## Competing interests

The authors declare that they have no competing interests.

## Authors’ contributions

VA, CT, JDi, PEH and JD planned, co-ordinated and conducted the study. VA, CT and MB are responsible for patient recruitment. ME, GS and BS perform CT and MRI scans and analysis. VA, CT, and MB perform planning and radiation therapy. ADS, LGS and UH perform ^18^ F-FMISO and ^18^ F-FDG investigations and analysis. Medical care and follow up is provided by VA and CT. Statistical analysis is performed by AKS. All authors read and approved the final manuscript.
